# Supplementary material for: Association between oral microbiome and seven types of cancers in East Asian population: a two-sample Mendelian randomization analysis
Source: Front Mol Biosci. 2023 Nov 21;10:1327893. doi: 10.3389/fmolb.2023.1327893 (PMC10702768; doi:10.3389/fmolb.2023.1327893)
Supplement: Supplementary file 1 [file DataSheet1.ZIP › Supplementary Materials/MR plots of saliva/saliva═╝/Gastric cancer/pheno.2065_to_gastric cancer_forest.pdf]

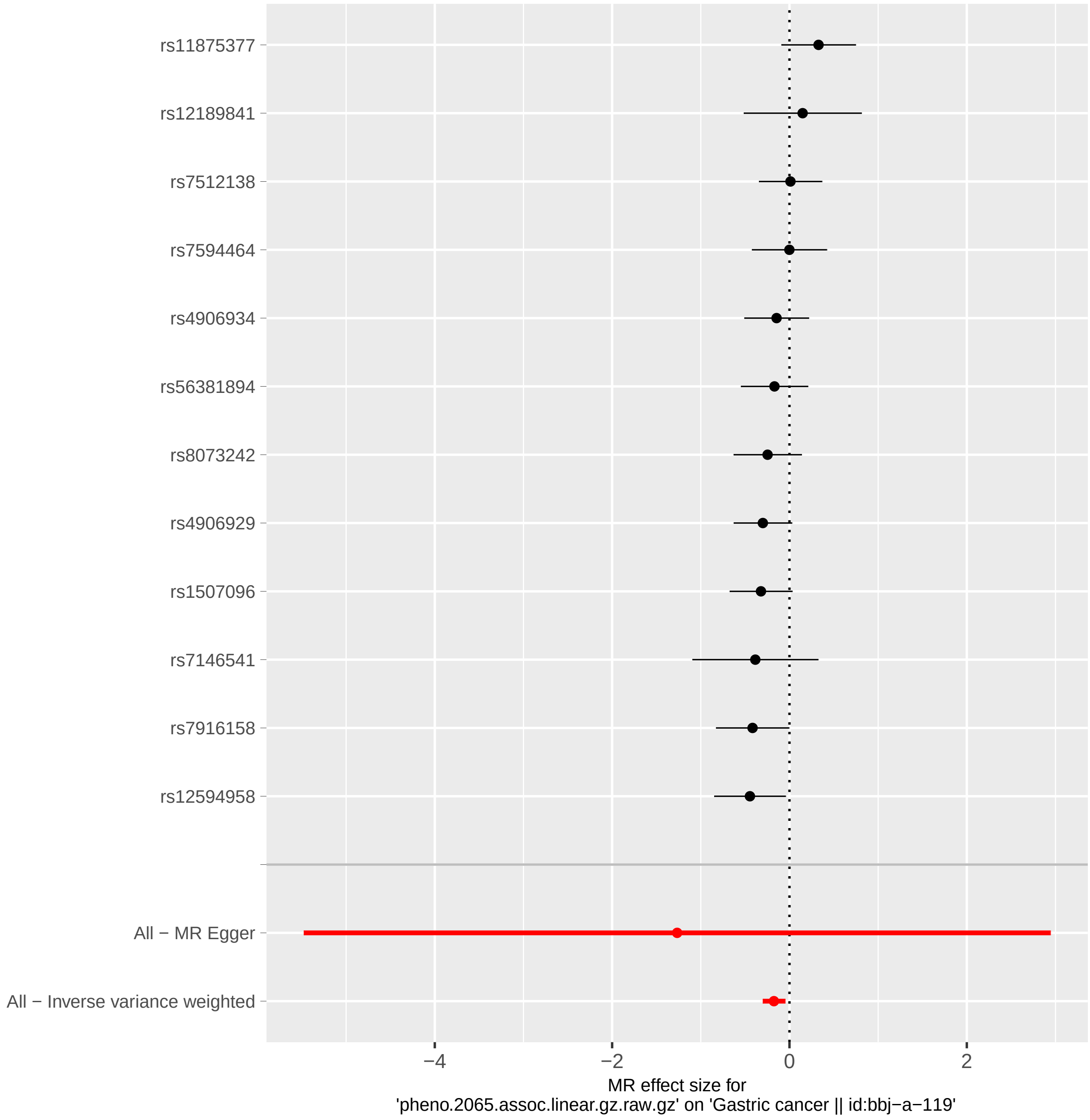

rs11875377
rs12189841
rs7512138
rs7594464
rs4906934
rs56381894
rs8073242
rs4906929
rs1507096
rs7146541
rs7916158
rs12594958
All − MR Egger
All − Inverse variance weighted
−4
−2
0
2
MR effect size for
'pheno.2065.assoc.linear.gz.raw.gz' on 'Gastric cancer || id:bbj−a−119'
